# Mediastinal Hepatoid Adenocarcinoma Treated With Arterial Interventional Therapy: A Case Report and Review of Literature

**DOI:** 10.3389/fonc.2022.785888

**Published:** 2022-04-19

**Authors:** Guiyuan Zhang, Chunyong Wen, Bin Chen, Haitao Dai, Run Lin, Yonghui Huang, Xianhong Xiang

**Affiliations:** Department of Radiology, The First Affiliated Hospital, Sun Yat-sen University, Guangzhou, China

**Keywords:** hepatoid adenocarcinoma, mediastinal, arterial infusion, interventional radiology, prognosis

## Abstract

Hepatoid adenocarcinoma (HAC) is an extremely rare extrahepatic carcinoma, which is pathologically featured by hepatocellular carcinoma (HCC) and marked by producing alpha-fetoprotein (AFP). HAC of mediastinum is extremely rare. For inoperable patients, the curative treatment options have not been established, and the outcome of HAC is usually poor. Here, we present a case of mediastinal HAC with normal serum AFP level who achieved well-controlled and good response after local–regional interventional approach combined with systemic PD-1 inhibitor. A 53-year-old male who complained of chest pain was admitted to our hospital in February 2021. A chest CT scan revealed several tumors in his mediastinum. The laboratory data showed normal serum AFP level. HAC was diagnosed through pathological assessment of biopsy. Surgery was not available due to the infiltration of sternum. Local regional FOLFOX chemotherapy was given by transarterial infusion, followed by transcatheter arterial chemoembolization, and thereafter combined with systemic anti-PD-1 treatment. The patient achieved favorable disease control and apparent symptom relief. So transarterial interventional therapy combined immunotherapy may be a possible and promising treatment for mediastinal HAC.

## Introduction

Hepatoid adenocarcinomas (HACs) belong to a rare group of extrahepatic tumors that show liver differentiation and usually produce alpha-fetoprotein (AFP), and this neoplasm is most frequently found in the stomach (1). Primary HACs in other anatomical locations, such as uterine bladder (2), ovaries (3), pancreas (4), and lungs (5), have been reported. Notably, to our best knowledge, only two cases of HAC in the mediastinum have been reported (6, 7). Primary HACs usually have poor prognosis because of frequent metastases at the early time of diagnosis. Given their rarity, no standard treatment or effective therapy has been established. An effective chemotherapy regimen is not available. The need for more effective treatment strategies is still urgent. The safety and effectiveness of arterial infusion chemotherapy have already been successfully evaluated for the treatment of several solid tumors (8–10). Immune checkpoint inhibitors (ICIs) targeting programmed cell death 1 (PD-1) pathways have made remarkable success in cancer treatment. To the best of our knowledge, hepatoid adenocarcinoma treated by interventional therapy has not been reported in English literature. Here, we report one patient of hepatoid adenocarcinoma in the mediastinum, whose serum AFP level was normal, treated with local regional chemotherapy using arterial interventional approach combined with a systemic PD-1 inhibitor.

## Case Presentation

**Figure 1:**
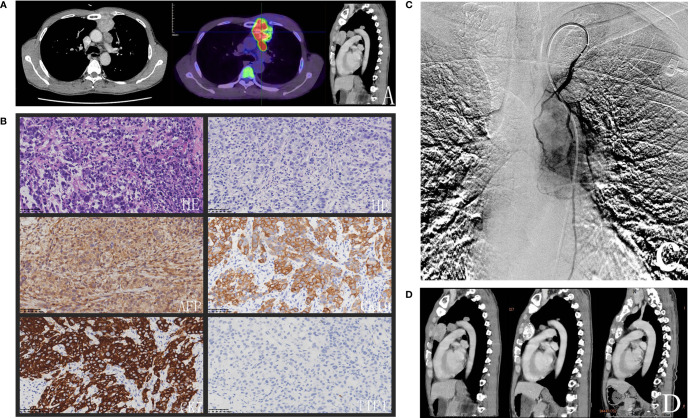
**(A)** Chest CT showed that three lesions in the mediastinum and the biggest lesion in the anterior mediastinum infiltrated sternum and FDG-PET/CT scan on 15 March 2021 revealed had abnormal uptake of FDG (SUVmax = 15.3). **(B)** The histopathologic examination showed that the rows and small nests of neoplastic cells were very pleomorphic, immunohistochemical staining of the tumor cells was shown subsequently. **(C)** Angiography illustrated tumors stained and the left internal thoracic artery was expanding and supplying blood of the tumors. **(D)** The size of the biggest lesion was minimized to 29 × 26 × 19 mm^3^ and the other lesions disappeared with ferroprotein decreased to 430.91 ug/L after four cycles of FOLFOX regimen arterial infusion combined with systemic PD-1 inhibitor every three weeks.

In February 2021, a 53-year-old man who had a smoking and drinking history of more than twenty years was admitted to our hospital with complaints that his front chest pain had lasted for half a year without breathlessness, wheeze, or stridor. There were no abnormalities of the chest shape, and any scars or skin deposits on the chest wall. The breath sound vesicular and no additional sounds, namely, crackles and pleural friction rub occurred. In no time, chest CT detected three soft tissue masses with irregular margins and mild heterogeneous enhancement in his mediastinum, the biggest one was of 59 × 39 × 26 mm^3^ in the anterior mediastinum and infiltrated the sternum, and the other lesions less than 22 mm located in the upper and lower mediastinum was close to the aorta (**Figure 1A**). However, most of his laboratory tests were normal, namely, blood cell counts, blood coagulation tests with tests of prothrombin time, and liver function tests. Regarding to tumor markers, serum level of AFP was normal (0.89 ug/L, normal range, 0–20.0 ug/L) and carcinoembryonic antigen (CEA) was slightly increased (5.08 ug/L, normal range, 0–5.0 ug/L), CA125 and CA199 were also normal. No abnormality was found in the hepatitis tests, namely, hepatitis B and hepatitis C. Initially, we diagnosed a likelihood of lung cancer, invasive thymoma or lymphoma.

In order to confirm the diagnosis, a percutaneous needle biopsy of the anterior mediastinal lesion was performed under CT guidance. The histopathologic examination revealed that the rows and small nests of neoplastic cells were very pleomorphic. (**Figure 1B**). By immunohistochemistry, the tumor cells stained positive for different cytokeratin antibodies (CK19, CK7, and CK20). HepPar1, focal AFP, and CD117 were also positive. Germ cell markers (CD30, OCT 3/4) along with other markers were negative, namely, CK5/6, TTF-1, NapsinA, TdT, Vimentin, P40, CD20, CD79a, CD3, CD5, Arginase-1, SALL4, P63, Syn, SATB2, CDX-2. Proliferative activity by Ki67 labeling index was high (70%). The pathologist drew a conclusion that it was consistent with the epithelial malignant tumor. Considering the hepatoid differentiation phenotype, it was necessary to distinguish the hepatoid adenocarcinoma from hepatocellular carcinoma metastasis. However, mediastinal metastases from a gastric hepatoid carcinoma or hepatocellular carcinoma were ruled out in this case soon, because no obvious gastric or liver tumors were apparent on the preoperative CT contrast-enhanced scan. HCCs are often associated with similar clinical context like cirrhosis, chronic hepatitis B or chronic hepatitis C infection. HAC of lungs have been reported with positive TTF-1 which was negative in the present case (11). An angiography also illustrated tumors stained and the left internal thoracic artery was expanding and supplying blood of the tumors (**Figure 1C**). The bronchial artery, the most common blood supply artery of pulmonary cancer, was normal. Taking these results together, the patient was diagnosed with HAC of mediastinum. Because of the invasion of the sternum and the close relationship to the aorta of multiple lesions, the patient was not suitable for surgical resection, and debulking was refused from the patient and his family for the risks. The lesions are relatively localized and obviously stained by the left internal thoracic artery. Therefore, a transcatheter chemoembolization of feeding artery of the tumor was performed with lipiodol-epirubicin farrago after the left internal thoracic artery had been prophylactic embolization using steel coils. Local–regional chemotherapy was applied to increase the drug concentration and reduce systemic toxicity. Transarterial infusion of palliative chemotherapy was administered with FOLFOX (oxaliplatin 85 mg/m^2^ on day 1; LV 200 mg/m^2^ on days 1 and 2; and FU 400 mg/m^2^ followed by 600 mg/m^2^ on days 1 and 2). A following FDG-PET/CT scan on 15 March 2021 revealed an enlargement of the biggest mass, which had abnormal uptake of FDG (SUVmax = 15.3). Meanwhile, PET/CT showed no lesion in the liver and stomach, except for the known mediastinal lesions. Moreover, there was an elevation of ferroprotein about 1,325.38 ug/L (normal range is 16.40–323.00 ug/L). The patient was treated with 200 mg of Sintilimab, the PD-1 inhibitor usually used in lymphoma and advanced non-small cell lung cancer.

The patient received additional four cycles of regional chemotherapy with FOLFOX4 regimen by the left internal thoracic artery infusion combined with systemic anti-PD-1 therapy every three weeks. The latest CT scan on 28 July 2021 showed that the size of the biggest lesion was minimized to 29 × 26 × 19 mm^3^ and the other lesions disappeared (**Figure 1D**). Ferroprotein has fallen to 430.91 ug/L. No significant adverse event was found, and the patient still remains alive and continues to follow up till 12 November 2021.

## Discussion

Hepatoid adenocarcinoma (HAC) is an uncommon tumor featured by mimicking hepatocellular carcinoma and frequently producing alpha-fetoprotein (AFP) (1). The morphological characteristics of HAC consist of two intertwined zones, one hepatoid zone is histologically described similar as hepatocellular carcinoma, and the other is adenocarcinomatous zones with hyper-differentiated adenocarcinomas. The polygonal tumor cells mimicked hepatocellular carcinoma cells histologically with clear, eosinophilic, abundant cytoplasm and prominent nuclei in hepatoid zone. A few hyaline globules also could be visible in the cytoplasm of tumor cells (12). The majority of biopsy material was composed of very pleomorphic cells in the present case.

HACs are a very heterogeneous group of tumors, concerning their immunological characteristics more than the various anatomical location of tumor invasion. HAC often occurs in extrahepatic organs, the most common site is the stomach (13), other organs, namely, uterine bladder (2), ovaries (3), pancreas (4), and lungs (5). Hepatoid adenocarcinoma of mediastinum is an extremely rare subtype and has the lowest incidence among all HAC (<0.5%) (1), of which only two cases were reported till now (6, 7). Conventional immunohistochemical staining could detect AFP in the cytoplasm, however, a fraction of HACs do not express AFP. Thus, AFP is not specific to HAC but more frequently used in HCC, teratoma and germ cell tumor. HepPar1 antibody is sensitive and specific for hepatocyte differentiation, but it is usually negative in metastatic HAC even the liver metastases (12, 14). Immunostaining for cytokeratin antibodies contributes to diagnosing HAC. The tumor cells in both HCC and HAC preserving the same parenchyma-typical cytokeratin pattern are always positive for CK8 and CK18 (15, 16). CK19 and CK20 were expressed in 100 and 44% of stomach HAC, while CK7 was more frequently positive in HAC of “ductal” organs, such as ovaries, gallbladder, and pancreas (16), this case of mediastinal HAC expresses the above. TTF-1 immunoblots were targeted to the cytoplasm in both hepatocellular carcinoma and pulmonary hepatoid adenocarcinoma (11), which was negative in this case.

HAC histopathologically and immunohistochemically resembles hepatocellular carcinoma. Recently, Gurzu (17) developed a hypothesis that the gastric type hepatocellular carcinoma, which shows cytoplasmic VSIG1 expression and hepatoid carcinoma derive from a common stem cell, the VSIG1A-positive foregut pluripotent endodermal cell. The hypothesis offers an explanation for the frequent occurrence of HAC in the foregut-derived organs, namely, the stomach, pancreas, lung, and mediastinum. Notably, it is difficult to distinguish HAC from ectopic hepatocellular carcinoma, which is another rare type of tumor arising from ectopic liver (18). Both common location and morphological features are similar. Pathologists could find hepatocellular differentiation with adenocarcinomas in HAC, however, it is still extremely difficult to distinguish the HCC and adenocarcinoma cells morphologically when tumor cells are poorly differentiated. Here, the key proof to distinguish HAC from EHCC is arginase-1, which is considered the most sensitive marker of HCC, especially in poorly differentiated HCC (19), while it was negative.

Even though HAC is characterized by AFP-producing, which also suggests a close relationship between HAC and HCC, elevated serum AFP levels are not always observed in HAC. Most HACs always have increased AFP levels as high as several thousand ng/ml, normal serum AFP-level cases derived from different organs were also reported (11, 20). The normal serum AFP level in this case also helped distinguish HAC from germ cell tumor. Additionally, elevation of serum ferroprotein levels was evident in this case. With the onset of treatment, the tumors shrank and reduced, serum ferroprotein level was decreased, which suggested serum ferroprotein be used as the markers of tumor response.

The prognosis for HAC is regarded as poor, metastases to regional lymph nodes and the liver usually occurred at the time of diagnosis due to its aggressive nature (21). The one-year survival rate of 83 cases with HAC in various metastatic sites was 55%, and median overall survival was 11 months (16). Due to its rarity and lack of bedside practice, no standard therapies, namely, surgery, chemotherapy, and radiotherapy, seems to exist for treating HAC. Like other adenocarcinomas, resection is the best way to deal with HAC in the early stage. However, when the diagnosis is confirmed, most patients are in the advanced stage and lost the surgery chance by virtue of its highly aggressive nature. Most cases accepted adjuvant chemotherapy, and the chemotherapy regimens were usually selected mainly based on their location, paclitaxel and carboplatin in gynecological HAC and mostly 5-fluorouracil (5-FU), cisplatin, and irinotecan in gastrointestinal HAC (16). Even it is recommended that intensive treatment can improve patients’ survival and patients always receive multimodal therapy comprising tumor debulking, chemoradiotherapy, clinical benefit is still poor (22). It is urgent clinical need to find newer therapeutic agents or targeted therapies to improve prognosis of patients.

In the current case, surgical resection was deemed inappropriate after the evaluation of thoracic surgeons. The reasons were as follows: the invasion of the sternum, the close relationship to the aorta, and debulking refused from the patient. Given the hypervascular nature of the tumor and no extramediastinal lesions, local-regional chemotherapy transarterial intervention was proposed as a palliative treatment, which is frequently used in tumors with such nature. Thus, transarterial chemoembolization and transarterial infusion chemotherapy were applied. To the best of our knowledge, HAC treated by TACE or TAIC has not been reported. The key strength of such treatments includes their ability to raise the effectiveness for target lesions and reduce the systemic side effects through regional drug delivery. It is reported that hepatic arterial infusion chemotherapy has been an effective treatment for advanced HCC in Asian countries (23). Since the therapeutic agent first passes through the liver, the organ involved in its final metabolism, it promises better disease control and fewer adverse events (23). In light of that HAC morphologically resembles hepatocellular carcinoma and the close relationship between them, the FOLFOX regimen was chosen. The systemic FOLFOX regimen has been demonstrated to be effective and safe for intestinal adenocarcinoma and HCC (24, 25). Furthermore, it has been proved effective in some cases of HAC. It has been reported that a young female HAC patient who obtained a complete response for more than three years received chemotherapy with FOLFOX after resection of the tumor (26). Another case of peritoneal HAC colliding with liposarcoma, neoadjuvant FOLFOX was followed by resection after six cycles of treatment for stable disease (27). Notably, FOLFOX has been reported to be applied in a case of colonic HAC as adjuvant treatment, in which the overall survival reached two years (28). The FOXAI study, which combines hepatic arterial infusion with FOLFOX, acquires a favorable survival rate, high response rate, acceptable toxicity for advanced HCC (29, 30).

The indicated immunotherapies of this rare tumor are lacking according to the current guidelines, but the potential response to immunotherapy was observed in several cases. Chen (31) reported a case of unresectable KRAS-positive pulmonary hepatoid adenocarcinoma that achieved disease control with Sintilimab after multiple lines of chemotherapy. Victor (32) also reported that hepatoid adenocarcinoma of the lung still responded to anti-PD-L1 therapy even without PD-L1 expression. Sintilimab, an ICI targeting PD-1, has been approved by the National Medical Products Administration of China as a third-line treatment for Hodgkin lymphoma and shows a satisfying anti-tumor effect and a better safety profile compared to nivolumab and pembrolizumab in Hodgkin lymphoma, natural killer/T cell lymphoma, and advanced non-small cell lung cancer (NSCLC) (33), which occur anatomically close to mediastinal HAC. In this study, the size of the biggest mass got significantly reduced and the other lesions completely disappeared after five cycles of the combination of local regional arterial infusion chemotherapy and systemic anti-PD-1 therapy. No previous cases have been reported about the clinical effects of local regional arterial infusion chemotherapy combined with systemic anti-PD-1 therapy. Here, arterial infusion with FOLFOX chemotherapy combined with systemic PD-1 inhibitor induced reduction of the tumor mass, relief of the symptoms and potential improvement of survival. It could attribute to the use of proper interventional oncological techniques and the potential of immunotherapy. But the efficacy of this new approach still needs to be further clarified by more detailed study in the future.

## Conclusion

In conclusion, HACs are a heterogeneous group of malignancies, not only due to the site of tumor infiltration but also with their immunophenotype. Primary mediastinal HAC is an extremely rare subtype during them, of which the beneficial treatment is still unclear, and the efficacy is not unsatisfied among the existing ones. It is worth noting here that arterial intervention therapy may be a potential treatment for HAC in line with our case. Nevertheless, further studies of HAC are urgently needed, including the diagnoses and treatments.

## Data Availability Statement

The original contributions presented in the study are included in the article/supplementary material. Further inquiries can be directed to the corresponding author.

## Ethics Statement

Written informed consent was obtained from the individual(s) for the publication of any potentially identifiable images or data included in this article.

## Author Contributions

Conception: GZ. Administrative support: XX and YH. Provision of study materials or patients: GZ and CW. Collection and assembly of data: GZ. Data analysis and interpretation: BC. All authors listed have made a substantial, direct, and intellectual contribution to the work and approved it for publication.

## Conflict of Interest

The authors declare that the research was conducted in the absence of any commercial or financial relationships that could be construed as a potential conflict of interest.

## Publisher’s Note

All claims expressed in this article are solely those of the authors and do not necessarily represent those of their affiliated organizations, or those of the publisher, the editors and the reviewers. Any product that may be evaluated in this article, or claim that may be made by its manufacturer, is not guaranteed or endorsed by the publisher.
